# KAMPNet: multi-source medical knowledge augmented medication prediction network with multi-level graph contrastive learning

**DOI:** 10.1186/s12911-023-02325-x

**Published:** 2023-10-30

**Authors:** Yang An, Haocheng Tang, Bo Jin, Yi Xu, Xiaopeng Wei

**Affiliations:** 1 School of Software, North University of China, No.3 Xueyuan Road, Jiancaoping District, 030051 Taiyuan, Shanxi China; 2 Institute of Automation Chinese Academy of Sciences, 95 Zhongguancun East Road, 100190 Beijing, China; 3 School of Innovation and Entrepreneurship, Dalian University of Technology, No.2 Linggong Road, Ganjingzi District, 116024 Dalian, Liaoning China; 4 School of Computer Science and Technology, Dalian University of Technology, No.2 Linggong Road, Ganjingzi District, 116024 Dalian, Liaoning China

**Keywords:** Electronic medical records, Medication prediction, Intelligent healthcare system, Multi-source medical knowledge, Graph contrastive learning

## Abstract

**Backgrounds:**

Predicting medications is a crucial task in intelligent healthcare systems, aiding doctors in making informed decisions based on electronic medical records (EMR). However, medication prediction faces challenges due to complex relations within heterogeneous medical data. Existing studies primarily focus on the supervised mining of hierarchical relations between homogeneous codes in medical ontology graphs, such as diagnosis codes. Few studies consider the valuable relations, including synergistic relations between medications, concurrent relations between diseases, and therapeutic relations between medications and diseases from historical EMR. This limitation restricts prediction performance and application scenarios.

**Methods:**

To address these limitations, we propose KAMPNet, a multi-sourced medical knowledge augmented medication prediction network. KAMPNet captures diverse relations between medical codes using a multi-level graph contrastive learning framework. Firstly, unsupervised graph contrastive learning with a graph attention network encoder captures implicit relations within homogeneous medical codes from the medical ontology graph, generating knowledge augmented medical code embedding vectors. Then, unsupervised graph contrastive learning with a weighted graph convolutional network encoder captures correlative relations between homogeneous or heterogeneous medical codes from the constructed medical codes relation graph, producing relation augmented medical code embedding vectors. Finally, the augmented medical code embedding vectors, along with supervised medical code embedding vectors, are fed into a sequential learning network to capture temporal relations of medical codes and predict medications for patients.

**Results:**

Experimental results on the public MIMIC-III dataset demonstrate the superior performance of our KAMPNet model over several baseline models, as measured by Jaccard, F1 score, and PR-AUC for medication prediction.

**Conclusions:**

Our KAMPNet model can effectively capture the valuable relations between medical codes inherent in multi-sourced medical knowledge using the proposed multi-level graph contrastive learning framework. Moreover, The multi-channel sequence learning network facilitates capturing temporal relations between medical codes, enabling comprehensive patient representations for downstream tasks such as medication prediction.

## Background

The availability of immense accumulation of electronic medical records (EMR) data, coupled with advancements in deep computational methods, has provided a solid foundation for intelligent healthcare applications, including disease risk prediction [1–3] and medication prediction task [4–6]. Among these applications, the prediction of medications for patients plays a crucial role in assisting doctors in making efficient clinical decisions, thereby enabling more time for doctor-patient communication and improving the quality of medical services. Thus, it will be conducive to improving the medical service quality. Consequently, there has been a growing demand for deep learning-based medication prediction models.

However, the majority of existing methods are not specifically tailored to address scenarios where multiple medical experts collaborate in joint consultations for patients. In such situations, various types of medical knowledge, including common-sense medical ontology and empirical medical knowledge derived from historical EMR data, are taken into consideration during the medication decision-making process. As a result, conventional methods often yield suboptimal performance in these complex decision-making scenarios. Therefore, effectively capturing the intricate and diverse relationships between medical codes from multi-source medical knowledge to enhance medication prediction becomes a highly challenging yet significant task.

**Fig. 1:**
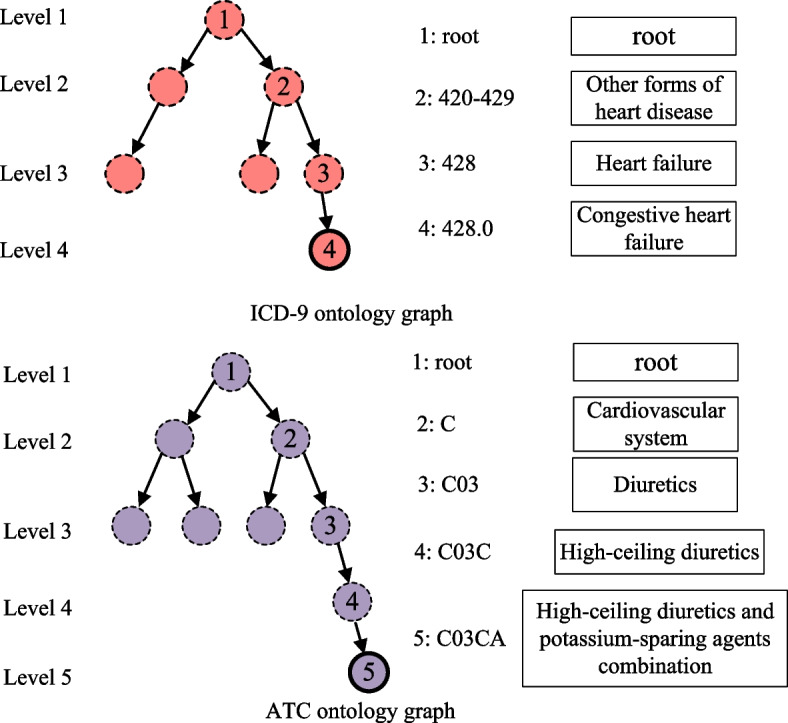
Illustration of Medical ontology graph: ICD-9 ontology for diagnosis and ATC ontology for medications. For each diagnosis or medication, we can obtain a unique parent path containing hierarchical ontology concepts from the root to the leaf levels. The ontology graph will be utilized to cultivate the inherent relations of homogeneous codes

As depicted in Fig. 1, the hierarchical structures inherent in the diagnosis ontology graph and medication ontology graph (representing common sense medical domain knowledge) imply relationships between homogeneous medical codes, which will contribute to the representation learning. The existing studies, such as GRAM [7] and KAME [8] utilize the diagnosis ontology graph to enhance the representations of diagnosis codes by incorporating information from relational medical codes using supervised methods. Meanwhile, Shang et al. [5] propose G-BIRT, combining a graph neural network and bidirectional encoder representation from transformers (BERT), to enhance medical code representations through a pre-training approach. These models consider the inherent relations between homogeneous medical codes in medical domain ontology graphs in a supervised or self-supervised manner to augment the medical code representations. Furthermore, MK-GNN [9] and COGNet [10] predominantly emphasize the incorporation of medical code relations, particularly between drug codes, using EHR graph or DDI graph. However, they tend to overlook concurrent relationships between diseases and the heterogeneous therapeutic relations between medications and diseases, as present in historical EMR data.

However, a limitation arises when attempting to transfer such forms of learned representations from one predictive task to another, such as from diagnosis prediction to medication prediction, despite using the same dataset. This necessitates repetitive model training to obtain medical code representations for each different downstream task. Moreover, purely supervised methods are ineffective in acquiring medical code representations when the downstream task is unknown. Therefore, there is an urgent need to develop a novel unsupervised method for learning the embeddings of medical codes based on the medical ontology graph. This approach would facilitate downstream tasks in diverse clinical scenarios, alleviating the limitations associated with the transferability and applicability of learned medical code representations.

Furthermore, the aforementioned models primarily focus on capturing the inherent relationships among homogeneous medical codes within the hierarchical structures of medical ontology graphs. However, they tend to overlook the valuable correlative relationships between both homogeneous and heterogeneous medical codes that are implicitly present in historical EMR data. This historical EMR data is typically regarded as a valuable source of empirical medical knowledge. For instance, clinicians commonly administer multiple medications simultaneously to patients to enhance the therapeutic effect, indicating the presence of synergistic relationships between medications. Additionally, major diseases frequently co-occur with inevitable concurrent diseases or symptoms, highlighting the concurrent relationships between diseases. Moreover, in prescriptions, medications are prescribed for specific diseases or symptoms, reflecting therapeutic causal relationships between medications and diseases. Unfortunately, only a limited number of studies have explicitly represented and captured these meaningful relationships hidden within EMR data for medication prediction.

**Fig. 2:**
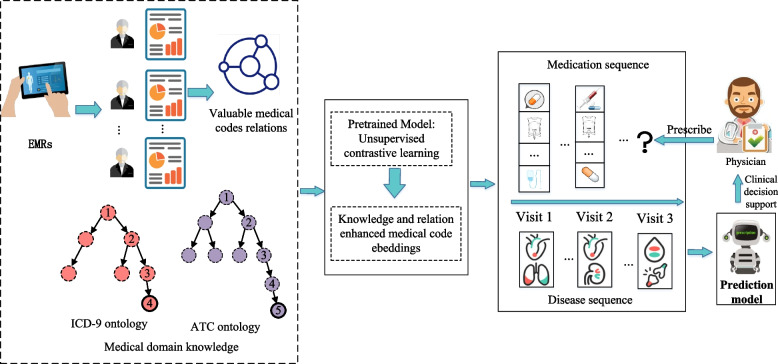
Main workflow of medication prediction using proposed model. It consists of three main steps: medical knowledge-related graph construction, unsupervised contrastive learning for medical code embeddings, and supervised sequential learning for medication prediction task

To address the aforementioned limitations and enhance the performance of medication prediction, we present a novel multi-sourced medical knowledge augmented network, named KAMPNet. The network leverages multi-level graph contrastive learning to capture the diverse relations between homogeneous or heterogeneous medical codes and improve their representations. The main workflow of medication prediction using the proposed model is depicted in Fig. 2: Firstly, similar to the existing method G-BIRT [5], we consider the inherent common-sense medical ontology graph (medical domain knowledge, shown in Fig. 1) to capture the local relations between medical codes. Additionally, we construct a medical codes relation graph, shown in Fig. 4, based on the co-occurrence of diagnosis codes and medication codes in a single visit from historical EMR data. This graph allows us to capture the global relations between medical codes. Secondly, to address the problems of label dependency and repetitive model training in existing ontology graph representation learning methods, we incorporate an improved graph contrastive learning framework based on DGI [11], which can provide better representations for downstream tasks without supervised labels and can facilitate to improve the model robustness [12]. This unsupervised learning approach enables us to obtain representations of medical codes from the multi-source knowledge graph. By infusing the information from relational medical codes, the augmented medical code representations are mutually enhanced, capturing both the local relations from the ontology graph and the global relations from the medical codes relation graph. Finally, the retrieved augmented medical code representations are fed into a supervised sequential learning model to capture the temporal relations between medical codes. This enables us to obtain a comprehensive patient representation, which can be utilized for medication prediction and assist in clinical decision-making.

In summary, the technical contributions of this paper are as follows: (1) We propose KAMPNet, a multi-source medical knowledge augmented medication prediction network, which incorporates a multi-level graph contrastive learning framework. To the best of our knowledge, our model is the first to capture valuable relations between medical codes and augment their representations using a cascaded unsupervised approach on both ontology graphs and constructed medical codes relation graph. (2) We integrate different graph encoders, such as the graph attention network and weighted graph convolutional network, into the multi-level graph contrastive learning framework to consider the meaningful relation weights between heterogeneous or homogeneous medical codes. (3) We present a sequential learning network that combines the multi-source embedding vectors of medical codes into the patient representation, enabling the capture of temporal relations between medical codes for medication prediction.

## Related works

### Deep learning in medication prediction

Medication prediction is a significant application of deep learning in intelligent healthcare systems, garnering considerable attention from researchers due to its practical importance. Researchers [4] have categorized medication prediction algorithms into instance-based and longitudinal sequential prediction methods.

Instance-based methods primarily focus on capturing the nonlinear relations between diagnosed disease status and the prescribed medications. For example, Zhang et al. [13] formulate the medication problem as a sequential decision-making problem and employ recurrent neural networks to encode the label dependency. Wang et al. [14] propose three linear models that combine multi-source patient information, including demographic data, laboratory indicators, and diagnosis outcomes, for personalized medication prediction. Wang et al. [15] transform the medication prediction task into an unordered Markov decision process, predicting prescription medications step by step. However, these methods overlook the critical temporal information present in historical EMR data, leading to suboptimal prediction performance.

Nowadays, longitudinal sequential prediction models that consider the temporal relations between historical medical records have become prevalent in medication prediction tasks. Jin et al. [16] present three different heterogeneous LSTM models to capture the interaction between heterogeneous temporal sequence data and incorporate two heterogeneous sequence information into the patient representation to predict the next stage of treatment medications. Shang et al. [4] incorporate sequential information, including diagnosis and procedure information, through multi-channel sequence learning networks to learn comprehensive patient representations for medication prediction. DMNC [17] and AMANet [6] integrate attention networks to capture the interactions between procedure and diagnosis sequences and model sequential dependencies. MeSIN [18] models both temporal dependencies between sequential medical records and the relations between hierarchical sequences for medication prediction. However, these models primarily focus on mining inherent relations between multiple medical sequences in EMR data and often neglect the empirical knowledge implicit in EMR data and external common-sense medical knowledge.

### Graph neural networks in healthcare applications

Graph neural networks (GNNs) [19] have emerged as effective frameworks for graph representation learning. GNNs leverage a neighbourhood aggregation mechanism to recursively aggregate and transform the representation vectors of adjacent nodes, effectively utilizing the topological relationships between graph nodes and enhancing the representation ability of nodes [20, 21]. As a result, GNNs have been widely used in biological and health informatics to model valuable relationships between multiple entities [4, 22–26]. The learned medical code embedding vectors in health informatics can be augmented to facilitate downstream predictive tasks. For example, GRAM [7] and KAME [8] leverage the diagnosis ontology graph to enhance the representations of diagnosis codes by incorporating information from relational diagnosis codes in the ontology graph using attention mechanisms. Zhang et al. [27] and Ye et al. [24] incorporate medical knowledge into sequential networks to enhance representation learning and obtain interpretable disease risk prediction results. Lu et al. [26] employ a patient-disease bipartite graph to create a weighted patient network (WPN) for learning robust patient representations in chronic disease prediction. Shang et al. [5] and Wang et al. [28] combine graph neural networks with bidirectional encoder representation from transformers (BERT) to capture inherent relationships between homogeneous medical codes in medical ontology graphs. Liu et al. [29] use temporal medical event graphs to represent complex relationships among different types of medical information for next-period prescription prediction. Mao et al. [30] construct medical graphs using historical EMR data to incorporate information from correlative entities into code representation for medication prediction and lab test imputation. Su et al. [31] construct dynamic co-occurrence graphs for each patient admission record and employ a graph-attention augmented sequential network to model inherent structural and temporal information for medication prediction. However, these models do not fully leverage the significant relations between medical codes due to a lack of global graph representation learning and the extraction of only local sub-graphs from the global guidance relation graph.

Although GNN-based models have achieved good performance in various tasks, they still face certain challenges. For instance, obtaining supervised labels can be complex and laborious, and models may require retraining for new tasks or feature changes. To address these issues, researchers have explored novel training methods for GNNs, such as graph self-supervised learning. Graph self-supervised learning is an unsupervised graph representation learning approach that relies solely on the topology and node information of the graph itself, without depending on explicit labels. For instance, Petar [11] incorporates Deep InfoMax [32] into the graph learning domain and models a general self-supervised learning framework based on the mutual information maximization. Similarly, Kaveh et al. [33] train the graph by maximizing the representation graph encoding of different graph structure perspectives. In the biomedical domain, Sun et al. [34] propose a novel molecular graph contrastive learning framework that incorporates local and global domain knowledge to enhance graph representation learning. Therefore, inspired by the advantages of contrastive learning methods, we incorporate a graph contrastive learning framework with two different graph encoders to learn the embeddings of medical codes from the medical ontology graph and medical codes relation graph in an unsupervised manner.

## Materials and methods

### Dataset and dataset preprocessing

To validate the effectiveness of the proposed KAMPNet, in this paper, we conduct the experiments on a publicly available dataset MIMIC-III [35] which is a large, freely-available database comprising deidentified health-related information associated with over 40,000 patients who were admitted to critical care units of the Beth Israel Deaconess Medical Center over the 12-year period between 2001 and 2012 and had relatively complete multi-sourced EMR. In particular, the dataset contains various kinds of heterogeneous information, such as diagnosed diseases, treatment medications and patient demographics etc., which satisfy the data requirement of our model. And we mainly extract useful information from tables PRESCRIPTIONS and DIAGNOSES_ICD.

**Fig. 3:**
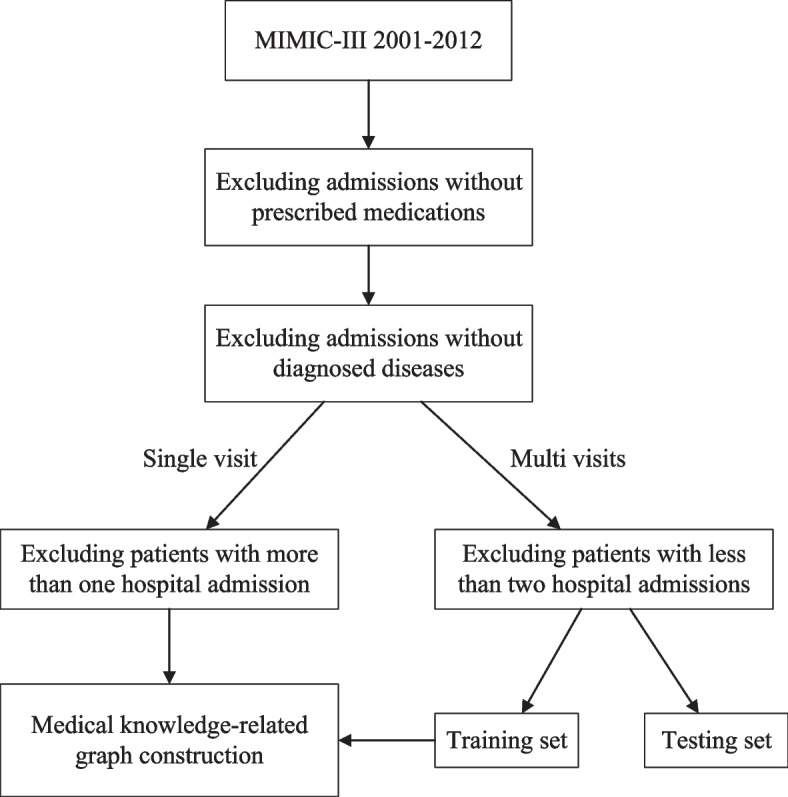
The flow chart of patients selection

The critical selection process of the experimental cohort is shown in Fig. 3. Firstly, the patients in the experimental cohort should have at least one complete visit record consisting of diagnosed diseases and treatment medications. Specifically, the patients with only one hospitalized visit record are mainly utilized to construct the medical knowledge graphs, including the ontology and relation graphs. While the patients with multiple hospitalized visit records are divided into training and testing sets and are harnessed for the sequence learning of KAMPNet. It is worth noting that the patients in the training set are also used to assist in constructing the multi-sourced medical knowledge graph.

Besides, similar to [4], the medications prescribed by doctors for each patient within the first 24 h are selected as the medication set since it belongs to a crucial period for each patient to get rapid and accurate treatment [36]. In addition, the medicine codes from NDC are transformed to ATC Level 3 for integration with MIMIC-III, and predicting category information not only guarantees the sufficient granularity of all the diagnoses but also improves the training speed, and predictive performance [7, 37]. Table 1 provides more information about the patient cohort from the dataset.

**Table 1:** Statistics of the MIMIC-III datasets

MIMIC III	Quantity
# of patients (Single visit)	30745
avg # of diagnosis	39
avg # of medication	52
# of unique diagnosis	1997
# of unique medication	323
# of patients (Multi visit)	6350
avg # of diagnosis	10.16
avg # of medication	7.33
avg # of visits	2.36
# of unique diagnosis	1958
# of unique medication	145

### Proposed model: KAMPNet

The proposed model KAMPNet mainly consists of three main substructures: medical knowledge-related graph construction, unsupervised contrastive learning for obtaining the enhanced medical code embeddings, and a supervised sequence learning network for medication prediction task.

#### Medical graph construction

##### Medical ontology graph construction

The hierarchical structures of the diagnosis classification system ICD-9 and medication classification system ATC imply the meaningful relations between medical codes, which have been constructed as the medical domain knowledge in previous studies [5, 7]. Similarly, the leaf nodes of the tree structure based graph comprise the medical codes of history EMR, and the non-leaf nodes mainly come from the medical codes classification system ICD-9 or ATC. By the above existing approach, the ICD-9 ontology graph $\mathcal{G}_d=\{\mathcal{V}_d,\mathcal{E}_d\}$ consisting of diagnosed disease codes and the hierarchical relations, the ATC ontology graph $\mathcal{G}_m=\{\mathcal{V}_m,\mathcal{E}_m\}$ consisting of treatment medication codes and the hierarchical relations are constructed respectively. And the unified indication of the above different medical ontology graphs is represented as $\mathcal{G}_{\ast} = \{\mathcal{V}_{\ast},\mathcal{E}_{\ast}\}$, where $\mathcal{V}_{\ast}$ is the set of graph nodes (medical codes), and $\mathcal{E}_{\ast}$ is the set of edges (the relations of medical codes). Among them, the set of medical codes $\mathcal{C}_{\ast}$ constitutes the leaf nodes of medical ontology graphs, and the set of all the graph nodes of $\mathcal{G}_{\ast}$ satisfies $\mathcal{V}_{\ast}= \mathcal{C}_{\ast} \cup \mathcal{C}^{\prime}$, where $\mathcal{C}^{\prime}$ denotes the set of non-leaf nodes.

##### Medical codes relation graph construction

In the actual clinical application scenario, patients will generally be diagnosed with a variety of diseases and given a variety of treatment medications during a single medical treatment, which also indirectly illustrates that there exist specific relations between diseases, medications, and between diseases and medications in the electronic medical records data. Therefore, based on the historical EMRs data generated by patients during each visit, and inspired by the dynamic weighted graph built on the history purchase records [38], we will construct the medical codes relation graph based on the co-occurrent medical codes including diagnosis codes and medication codes.

**Fig. 4:**
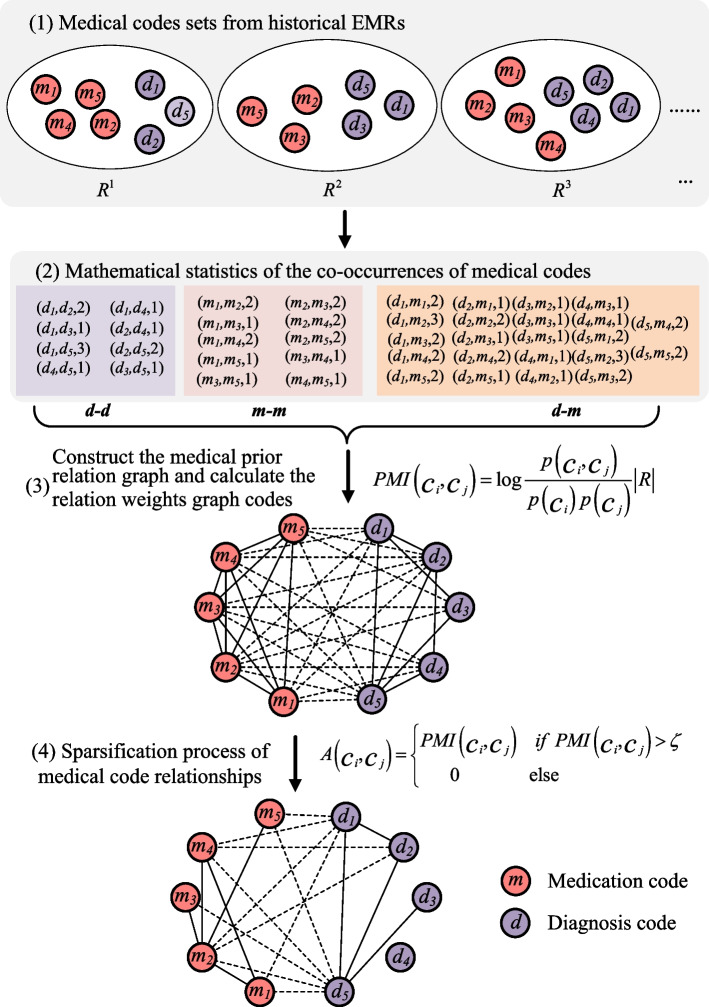
Construction process of medical codes relation graph

Figure 4 shows the detailed process of the medical codes relation graph construction based on the history EMR of patients. Since the patient with a single hospitalized visit has only one visit record, and the patient with multiple hospitalized visits has multiple visit records, in this paper, the construction of the medical codes relation graph does not consider the temporal sequence information but only considers the implicit relation between medical codes of history EMR data. Therefore, the visit-based medical record extracted from the patient’s history EMR is represented as $\varvec{R}^{i}$, and every hospitalized visit could produce a diagnosis code and medication code. As shown in Fig. 4, there are three historical records: $\varvec{R}^{1},\varvec{R}^{2},\varvec{R}^{3}$, and each record $\varvec{R}^{i}$ includes diagnosis code $m_{\ast}$ and medication code $d_{\ast}$. Based on the visit records shown in Fig. 4 (1), the corresponding medical code pairs in each visit record can be generated such as $(d_1,d_2)$, $(d_1,m_1)$, $(m_1,m_2)$, $\dots$. After mathematical statistics, the generated medical code pairs and the number of co-occurrence are shown in Fig. 4 (2). There are three implicit relations: the concurrent relation between diseases $d-d$, the synergistic relation between medications $m-m$, and the therapeutic relation between diseases and medication $d-m$. Then, considering that the relations between medical codes are not only related to the number of co-occurrence but also related to the frequency of medical codes, the pointwise mutual information (PMI) [39] commonly used in natural language processing to measure the relevance of words is introduced to calculate the relation weights between medical codes:

1 $$\begin{aligned} PMI(c_i,c_j)&=\log \frac{P(c_i,c_j)}{P(c_i)P(c_j)}\nonumber \\&=\log \frac{\frac{p(c_i,c_j)}{|\varvec{R}|}}{\frac{p(c_i)}{|\varvec{R}|}\frac{p(c_j)}{|\varvec{R}|}}\nonumber \\&=\log \frac{p(c_i,c_j)}{p(c_i)p(c_j)}|\varvec{R}|, \end{aligned}$$

where $|\varvec{R}|$ denotes the total number of visit records of all patients in the training dataset, $P(c_i,c_j)$ and $p(c_i,c_j)$ respectively denotes the probability and number of co-occurrence of medical codes $c_i$ and $c_j$ in single visit record, $P(c_i)$ and $p(c_i)$ indicates the probability and number of occurrence of medical code $c_i$ in visit records, $P(c_j)$ and $p(c_j)$ indicates the probability and number of occurrence of medical code $c_j$ in visit records. The mutual information between the above medical code pairs is for all the code pairs, including $d-d$,$m-m$ and $d-m$ forms, without distinguishing whether they are homogeneous medical codes or heterogeneous medical codes. The main reason is that the relation degree between the medical code pairs is obtained by calculating statistical mutual information, which belongs to the scope of quantitative analysis without involving any medical background. Therefore, the qualitative relation is ignored in the quantitative calculation, even though the heterogeneity between medical codes does exist in reality.

Then, as shown in Fig. 4 (3), the medical codes relation graph $\mathcal{G}_r = \{\mathcal{V}_r,\mathcal{E}_r,\mathcal{W}_r\}$ can be built through the triplets obtained by the previous step, where $\mathcal{V}_r$ represents the graph nodes set, also called the medical codes set $\mathcal{C} = [\mathcal{C}_d,\mathcal{C}_m]$, $\mathcal{E}_r$ is the graph edges set, $\mathcal{W}_r$ represents the relation weight. Moreover, the heterogeneity between diagnosis code and medication code would be neglected for the reason that the relation strength between medical codes is the critical consideration factor in this paper. The medical code pairs obtained in Fig. 4 (2) determine the edges between nodes in the relation graph. Thus, the edge weights in adjacency matrix $\varvec{A}$ of the medical codes relation graph $\mathcal{G}_r$ can be further calculated as follows:

2 $$\begin{aligned} A(c_i,c_j)= \left\{ \begin{array}{ll} PMI(c_i,c_j) &{}\text {if } PMI(c_i,c_j)> \zeta , \\ 0 &{} \text {else}. \end{array}\right. \end{aligned}$$

Different from the calculation method in [31], here, we incorporate a graph sparsity factor $0<\zeta <PMI_{max}$ in Eq. (2), which aims to mitigate the effects of noise that might be introduced by relying solely on statistical quantitative computation method and ignoring medical expertise. Moreover, the relation graph belongs to a symmetric matrix, i.e., $A(c_j,c_i)=A(c_i,c_j)$. In this way, the ultimately complete medical codes relation graph is obtained as shown in Fig. 4 (4) In the end, another initial representation method of medical codes relation graph $\mathcal{G}_r$ can be obtained, i.e., $\mathcal{G}_r=(\mathcal{C},\varvec{A})$.

#### Unsupervised contrastive learning on medical ontology graphs

To avoid the dependency on the labels and make the learned medical codes representations directly applied in the downstream tasks such as medication prediction, the unsupervised graph contrastive learning method based on DGI [11] are incorporated to learn the medical codes representations by maximizing the mutual information of graph representation in the following two cascaded sections.

**Fig. 5:**
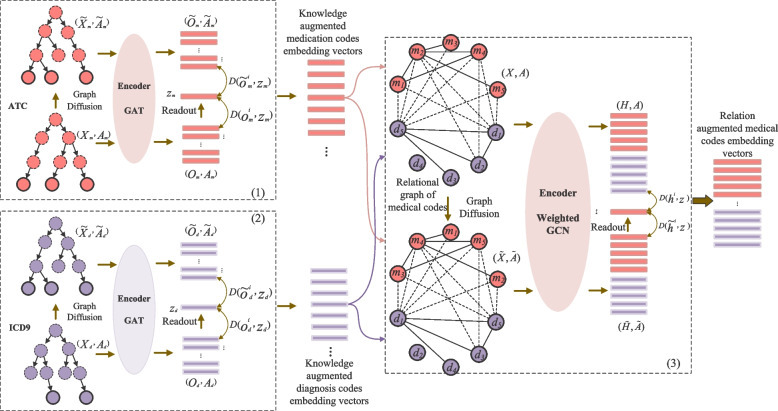
Multi-level unsupervised contrastive learning framework. It consists of three different contrastive learning processes: (1) Contrastive learning on medication code ontology graph; (2) Contrastive learning on diagnosis code ontology graph; (3) Contrastive learning on medical codes relation graph

As illustrated in Fig. 5, (1) and (2) respectively describe the introduced unsupervised contrastive learning processes on the medication code ontology graph and diagnosis code ontology graph. Such a learning process consists of four critical substructures: graph data augmentation, GNN-based encoder, graph pooling layer, and contrastive loss function.

Similar to previous knowledge-enhanced methods presented in G-BIRT [5], the medical codes $c_i$ of ontology graph $\mathcal{G}_{\ast}$ can be randomly assigned an initialized embedding vector $\varvec{v}_i$ which could be optimized and updated through a learned embedding matrix $\varvec{W}_e\in \mathbb {R}^{\mathcal{C}_{\ast}\times {d}}$, where *d* indicates the dimension of the medical code embedding vector. Then the constructed medical ontology graph $\mathcal{G}_{\ast} = \{\mathcal{V}_{\ast},\mathcal{E}_{\ast}\}$ can be indicated as $(\varvec{X}_{\ast},\varvec{A}_{\ast})$, where $\varvec{X}_{\ast}$ represents the set of initialized medical codes representations of the ontology graph $\mathcal{G}_{\ast}$ and $\varvec{A}_{\ast}$ represents the corresponding adjacency matrix.

##### Graph data augmentation

Then as shown in Fig. 5 (1) and (2), the initialized constructed medical ontology graph $(\varvec{X}_{\ast},\varvec{A}_{\ast})$ undergoes graph data augmentation to obtain the negative sample via corruption function such as the graph nodes permutation. In detail, the $\mathcal{C}$ is used to randomly perturb the nodes without changing the topology structure of the ontology graph; namely, the adjacency matrix $\varvec{A}_{\ast}$, which aims to obtain the augmented ontology graph: $(\hat{\varvec{X}}_{\ast},\hat{\varvec{A}}_{\ast})=\mathcal{C}(\varvec{X}_{\ast},\varvec{A}_{\ast})$.

##### GNN-based encoder

In fact, there should be no restrictions on the choice of graph encoders. Here, to fully utilize the inherent topological hierarchical structure of the medical ontology graph for further capturing the implicit relations between medical codes, we directly incorporate the graph attention network (GAT) [40] used in previous study G-BIRT [5] as the graph encoder $\mathcal{F}$ to obtain the medical codes embedding representations of medical ontology graphs.

Given the medical ontology graph $\mathcal{G}_{\ast}$ is a hierarchical structural graph with a parent-child substructure, the relation between medical codes can be captured from two different paths. That is, on the one hand, the medical codes corresponding to the parent nodes should infuse the information corresponding to the medical code of the child node; on the other hand, the embedding representation information corresponding to the medical code of the parent node also should be transmitted to the leaf node. For each non-leaf node in the medical domain knowledge graph $c^{\prime}\in \mathcal{C}^{\prime}$ and the leaf node $c_{\ast}\in \mathcal{C}_{\ast}$, the corresponding augmented embedding representations $\varvec{v}_{c_{\ast}}\in \mathbb {R}^d$ and $\varvec{h}_{o_{\ast}}\in \mathbb {R}^d$ can be computed as follows:

3 $$\begin{aligned} \varvec{v}_{c^{\prime}}&= f(c^{\prime},ch(c^{\prime}),\varvec{W}_e),\nonumber \\ \varvec{o}_{c_{\ast}}&= f(c_{\ast},pa(c_{\ast}),\varvec{V}_e), \end{aligned}$$

where $f(\cdot ,\cdot ,\cdot )$ indicates the graph information aggregation function, $ch(c^{\prime})$ is a function to extract all direct child nodes of non-leaf medical code $c^{\prime}$, while $pa(c_{\ast})$ represents a function which can extract all parent nodes of the leaf medical code $c_{\ast}$. The above method realizes the two-way information transmission (from top to bottom and from bottom to top) through the hierarchical structure. In this way, the implicit relations between the medical codes can be fully captured; that is, the medical codes representations are augmented, which can further alleviate the sudden decline of prediction accuracy caused by the insufficient learning problems of the tail codes in electronic medical records.

Due to the graph information aggregation in above two-way information transmission process requires considering the relation difference between medical codes, in this paper, we incorporate the graph attention network (GAT) [40] as the aggregation function $f(\cdot ,\cdot ,\cdot )$, and it is also the shared graph encoder of the graph contrastive learning framework for calculating the graph nodes representations. Specifically, the representation vectors of each medical code $c_i$ is computed using the graph attention network aggregation function $f(\cdot ,\cdot ,\cdot )$ as follows:

4 $$\begin{aligned} f\left( c_{i}, p\left( c_{i}\right) , \varvec{H}_{e}\right) =\Vert _{k=1}^{K} \sigma \left( \sum _{j \in \mathcal{N}_i} \alpha _{i, j}^{k} \varvec{W}^{k} \varvec{v}_{j}\right) , \end{aligned}$$

where $\mathcal{N}_i = \{c_{i}\} \cup pa\left( c_{i}\right)$ denotes the first order neighborhood nodes set of medical code $c_i$ in the medical ontology graph, $\Vert$ is the concatenation operation of the embedding representations computed by multi-head attention, *K* is the number of attention heads, $\sigma$ is the nonlinear activation function Sigmoid, $\varvec{W}^{k}\in \mathbb {R}^{m \times d}$ is the transformation matrix to be learned, where $m=d/k$. While, $\alpha _{i, j}^{k}$ indicates the *k*-th standardized relevance score, which can be calculated as follows:

5 $$\begin{aligned} \alpha _{i, j}^{k}=\frac{e^{\left( \text {LeakyReLU}\left( \varvec{a}^{\textrm{T}}\left[ \varvec{W}^{k} \varvec{v}_{i} \Vert \varvec{W}^{k} \varvec{v}_{j}\right] \right) \right) }}{\sum _{k \in \mathcal{N}_{i}} e^{\left( \text {LeakyReLU}\left( \varvec{a}^{\varvec{\top }}\left[ \varvec{W}^{k} \varvec{v}_{i} \Vert \varvec{W}^{k} \varvec{v}_{k}\right] \right) \right) }} \end{aligned}$$

where $\varvec{a}\in \mathcal{R}^{2m}$ is the learned weight parameter. And we propose to use $\text {LeakyReLU}$ [41] as the nonlinear activation function. The reason is Leaky ReLU maintains a small slope (typically a small positive value, such as 0.01) for negative inputs, unlike traditional ReLU which has a gradient of zero for negative inputs and the occurrence of "dead" or "dying" neurons. This non-zero gradient property is beneficial as it provides a continuous and differentiable activation function, allowing gradient-based optimization methods to be applied effectively during backpropagation. The continuous gradient enables smoother and more stable learning, aiding convergence and improving the overall training process.

Therefore, we can utilize the above graph encoder $\mathcal{F}$ to respectively compute the embedding representations of the medical codes from medical ontology graph $\mathcal{G}_{\ast}$ and augmented medical ontology graph $\hat{\mathcal{G}}_{\ast}$ as follows:

6 $$\begin{aligned} \varvec{O}_{c_{\ast}}&= \mathcal{F}(\mathcal{V}_{\ast},\mathcal{E}_{\ast})=\{\varvec{o}_{c_{\ast}^1},\varvec{o}_{c_{\ast}^2},...,\varvec{o}_{c_{\ast}^{|\mathcal{C}|}}\},\nonumber \\ \hat{\varvec{O}_{c_{\ast}}}&=\mathcal{F}(\hat{\mathcal{V}}_{\ast},\hat{\mathcal{E}}_{\ast})=\{\hat{\varvec{o}}_{c_{\ast}^1},\hat{\varvec{o}}_{c_{\ast}^2},...,\hat{\varvec{o}}_{c_{\ast}^{|\mathcal{C}|}}\}, \end{aligned}$$

where $\varvec{O}_{c_{\ast}}$ and $\hat{\varvec{O}_{c_{\ast}}}$ respectively denotes the embedding representations sets of medical codes from $\mathcal{G}_{\ast}$ and $\hat{\mathcal{G}}_{\ast}$.

##### Graph pooling layer

The graph pooling layer is mainly leveraged to compute the global feature vector $\varvec{z}_{\ast}$ through $\text {readout}$ function $\mathcal{R}$:

7 $$\begin{aligned} \varvec{z}_{*}=\mathcal{R}(\varvec{O}_{c_{\ast}})=\frac{1}{|\mathcal{C}_{\ast}|}\sum _{i=1}^{|\mathcal{C}_{\ast}|}\varvec{o}_{c_{\ast}^i}, \end{aligned}$$

where $\varvec{O}$ indicates the unified symbol of embedding representation of medical codes of the medical ontology graph (diagnosis and medication).

##### Contrastive loss function

In order to train the graph encoder end-to-end and learn to obtain the informative medical codes embedding vectors and the medical ontology graph embedding representation, we still utilize the maximization of mutual information [32] between medical code embedding vector $\varvec{o}_{c_{\ast}^i}$ and the medical ontology graph embedding representation $\varvec{z}_{\ast}$ as the objective loss function. First, the negative and positive sample pairs $(\varvec{o}_{c_{\ast}^i},\varvec{z}_{\ast})$ and $(\hat{\varvec{o}}_{c_{\ast}^i},\varvec{z}_{\ast})$ can be obtained through graph encoder and graph pooling layer. Then, the discriminator $\mathcal{D}$ is introduced to score the positive and negative sample pairs:

8 $$\begin{aligned} \mathcal{D}(\varvec{o}_{c_{\ast}^i},\varvec{z}_{\ast})&=\sigma ({\varvec{z}_{\ast}}^T\varvec{W}_{D}\varvec{o}_{c_{\ast}^i} ),\nonumber \\ \mathcal{D}(\hat{\varvec{o}}_{c_{\ast}^i},\varvec{z}_{\ast})&=\sigma ({\varvec{z}_{\ast}}^T\varvec{W}_{D}\hat{\varvec{o}}_{c_{\ast}^i}). \end{aligned}$$

Finally, the overall objective function is to maximize the mutual information in the form of JS divergence as follows:

9 $$\begin{aligned} \mathcal{L}&=\frac{1}{|\mathcal{C}_{\ast}|}\sum _{i=1}^{|\mathcal{C}_{\ast}|} \mathbb {E}_{(\mathcal{V}, \mathcal{E})}\left[ \log \mathcal{D}\left( \varvec{o}_{c_{\ast}^i},\varvec{z}_{\ast}\right) \right] \nonumber \\&\quad +\frac{1}{|\mathcal{C}_{\ast}|}\sum _{j=1}^{|\mathcal{C}_{\ast}|} \mathbb {E}_{(\tilde{\mathcal{V}}, \tilde{\mathcal{E}})}\left[ \log \left( 1-\mathcal{D}\left( \hat{\varvec{o}}_{c_{\ast}^i},\varvec{z}_{\ast}\right) \right) \right] \end{aligned}$$

Through the above detailed graph contrastive learning on medical ontology graphs, we can obtain the knowledge augmented medical codes embedding vectors $\varvec{O}_{c}$ including knowledge augmented medication codes embedding vectors $\varvec{O}_{c_m}$ and knowledge augmented diagnosis codes embedding vectors $\varvec{O}_{c_d}$ (as shown in Fig. 5).

#### Unsupervised contrastive learning on medical relation graph

Factually, the obtained medical codes embedding vectors have infused the relational information from correlative medical codes, which can provide the initial embedding vectors for the nodes of the medical codes relation graph in this section. However, the medical ontology graphs do not explicitly provide the practically meaningful relations between medical codes and the implicit relations between homogeneous medical codes in the hierarchical topology structure (belonging to a local relation). Therefore, in this section, the medical codes relation graph constructed on the basis of history EMR can directly models the explicit relations (belonging to a global relation) between homogeneous and heterogeneous medical codes through quantitative statistics and then uses the graph contrastive learning framework shown in Fig. 5 (3) to learn the relation augmented embedding vectors of the medical codes of the medical codes relation graph. The learning process will fully leverage the global relation between medical codes to model the interrelations of the medical codes embedding vectors.

As shown in Fig. 5 (3), the unsupervised contrastive learning on medical codes relation graph also includes four critical steps. First, the initialization vector of the corresponding node of medical codes from the medical codes relation graph can be retrieved directly from the obtained knowledge augmented medical code embedding vectors set $\varvec{O}_{c}$, and denoted as $\varvec{X}\subset \varvec{O}_{c}$. Then, the medical codes relation graph can be indicated as $\mathcal{G}_r=(\mathcal{C},\varvec{A}):(\varvec{X},\varvec{A})$. Subsequently, we can obtain the negative graph sample through graph nodes perturbation, namely augmented medical codes relation graph $(\widetilde{\varvec{X}},\widetilde{\varvec{A}})\backsim (\varvec{X},\varvec{A})$. After that, the incorporated shared graph encoder is incorporated to encode the above two relation graphs. Different from the selected graph encoder GAT, the specially weighted graph convolutional network (GCN) [20] is incorporated as the graph encoder in the graph contrastive learning framework in this section to infuse the relation weights between medical codes. Taking the medical codes relation graph $(\varvec{X},\varvec{A})$ as an example, we calculate the corresponding embedding vectors as follows:

10 $$\begin{aligned} \varvec{H}=\hat{\varvec{D}}^{-1/2}\hat{\varvec{A}}\hat{\varvec{D}}^{-1/2}\varvec{X}\Theta \end{aligned}$$

where $\hat{\varvec{A}}=\varvec{A}+\varvec{I}$,$\varvec{I}$ is the identity matrix that aims to avoid the information loss caused by the small number of neighborhood nodes. The diagonal matrix $\hat{\varvec{D}}_{ii} = \sum _{j=0}A_{ij}$ is used to normalize the weights of the connected edges of each node from the relation graph according to the edge weights from the adjacency matrix $\hat{\varvec{A}}$. $\Theta \in \mathbb {R}^{d_x \times d_h}$ is the learned network parameter. Considering the edge weights, the weighted graph encoder realizes the mutual infusion of correlative medical codes information in the medical codes embedding vectors learning process according to the relevance degree. Similarly, with the help of the above graph encoder weighted GCN, we can further encode the negative graph sample, i.e., augmented medical codes relation graph $(\widetilde{\varvec{X}}, \widetilde{\varvec{A}})$, and obtain the corresponding medical codes embedding matrix $(\widetilde{\varvec{H}},\widetilde{\varvec{A}})$ (shown in Fig. 5 (3)). Afterwards, the $\text {readout}$ function $\hat{\mathcal{R}}$ is used to compute the global embedding vector $\varvec{z}$ of medical codes relation graph $\varvec{H}={h_1,h_2,\dots ,h_{|\mathcal{C}|}}$:

11 $$\begin{aligned} \varvec{z} = \hat{\mathcal{R}}(\varvec{O}_{c_{*}})=\frac{1}{|\mathcal{C}|}\sum _{i=1}^{|\mathcal{C}|}\varvec{h}_i \end{aligned}$$

Finally, the constructed objective function based on the discriminant equation is introduced to optimize the graph contrastive learning process for the medical codes relation graph. Similar to the contrastive learning framework for medical ontology graph, we still utilize the maximization of mutual information [32] between the embedding representation vector $\varvec{h}_i$ of medical code on the medical codes relation graph and the global relation graph embedding vector $\varvec{z}$. Firstly, the negative and positive sample pairs $(\varvec{h}_{i},\varvec{z})$ and $(\hat{\varvec{h}}_{i},\varvec{z})$ can be obtained through graph encoder and graph pooling layer. Then, the discriminator $\mathcal{D}$ is introduced to score the positive and negative sample pairs:

12 $$\begin{aligned} \mathcal{D}(\varvec{h}_{i},\varvec{z})&=\sigma ({\varvec{z}}^T\varvec{W}_{D}\varvec{h}_{i} )\nonumber \\ \mathcal{D}(\hat{\varvec{h}}_{i},\varvec{z})&=\sigma ({\varvec{z}}^T\varvec{W}_{D}\hat{\varvec{h}}_{i}). \end{aligned}$$

Then, the overall objective function is to maximize the mutual information in the form of JS divergence as follows:

13 $$\begin{aligned} \mathcal{L}&=\frac{1}{|\mathcal{C}|}\sum _{i=1}^{|\mathcal{C}|} \mathbb {E}_{(\varvec{X}, \varvec{A})}\left[ \log \mathcal{D}\left( \varvec{h}_{i},\varvec{z}\right) \right] \nonumber \\&\quad +\frac{1}{|\mathcal{C}|}\sum _{j=1}^{|\mathcal{C}|} \mathbb {E}_{(\widetilde{\varvec{X}}, \widetilde{\varvec{A}})}\left[ \log \left( 1-\mathcal{D}\left( \hat{\varvec{h}}_{i},\varvec{z}\right) \right) \right] . \end{aligned}$$

After continuous optimization and iterative calculation, the final relation augmented medical codes embedding representation vectors as shown in Fig. 5 (3) can be obtained, which, in the end, not only integrates the information from globally correlative heterogeneous and homogeneous medical codes embedding vectors in the medical codes relation graph but also infuses the information from locally correlative homogeneous medical codes embedding vectors implied in the medical domain knowledge graphs (the medical ontology graphs).

#### Sequential learning network for medication prediction

**Fig. 6:**
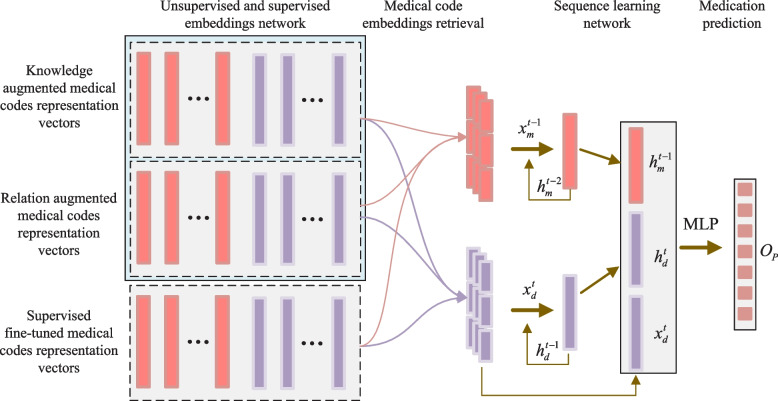
Medication prediction framework

As illustrated in Fig. 6, the sequential medication prediction framework comprise three critical substructures, i.e., multi-sourced medical codes are embedding vectors fusion, sequence learning network, and the comprehensive medication prediction.

##### Multi-sourced medical codes embedding vectors fusion

The knowledge augmented medical codes embedding vectors $\varvec{O}_{c} = \{\varvec{O}_{c_m},\varvec{O}_{c_d}\}$ and the relation augmented medical codes embedding vectors $\varvec{H}$ are respectively obtained through corresponding graph contrastive learning network. While for each specific prediction task, it has different task-specific requirements for the medical codes embedding vectors. Therefore, in the medication prediction task, the learned medical codes embedding representation vector $\varvec{e}^i\in \mathbb {R}^d$ that can be supervised and fine-tuned in the specific prediction task is also incorporated:

14 $$\begin{aligned} \varvec{e}^i = \varvec{W}^{e}\varvec{c}^i, \end{aligned}$$

where $\varvec{W}^{e} \in \mathbb {R}^{|\mathcal{C}|\times d}$ is the medical codes embedding matrix to be learned, $\mathcal{C} = [\mathcal{C}_d,\mathcal{C}_m]$ is the union set of medical codes, and $\mathcal{C}_d$ and $\mathcal{C}_m$ respectively denote the sets of diagnosis codes and medication codes. In this way, we can get the dense embedding vector matrix of medical codes that can be learned, i.e. $\varvec{E} = [\varvec{e}^1,\dots ,\varvec{e}^{|\mathcal{C}|}] \in \mathbb {R}^{|\mathcal{C}|\times d}$.

Each hospitalized visit will generate different medical codes corresponding to different diagnoses and medications. The corresponding medical codes embedding vectors could be retrieved from the obtained medical codes embedding vectors sets, including knowledge augmented medical codes embedding vectors set $\varvec{O}_{c}$, relation augmented medical codes embedding vectors set $\varvec{H}$, and the supervised learning medical codes embedding vectors set $\varvec{E}$. For instance, we can first retrieve the corresponding embedding representations of medical codes produced in *i*-th visit record from the medical codes embedding vectors sets, i.e. the medical diagnosis codes embedding representations including $\varvec{O}^i_{d}\subset \varvec{O}_{c}$, $\varvec{H}^i_{d}\subset \varvec{H}$ and $\varvec{E}^i_{d}\subset \varvec{E}$, the medical medication codes embedding representations including $\varvec{O}^i_{m}\subset \varvec{O}_{c}$, $\varvec{H}^i_{m}\subset \varvec{H}$ and $\varvec{E}^i_{m}\subset \varvec{E}$. Then the corresponding mean values of medical codes representation vectors sets are calculated to obtain the visit-level diagnosis codes embedding vectors containing $\varvec{o}^i_d$, $\varvec{h}^i_d$ and $\varvec{e}^i_d$, and medication codes embedding vectors containing $\varvec{o}^i_m$, $\varvec{h}^i_m$ and $\varvec{e}^i_m$; Finally, the calculated embedding vectors are respectively concatenated together to obtain the input of diagnosis codes sequence learning network and medication sequence learning network, i.e. $\varvec{x}^i_d = [\varvec{o}^i_d,\varvec{h}^i_d,\varvec{e}^i_d]$ and $\varvec{x}^i_m = [\varvec{o}^i_m,\varvec{h}^i_m,\varvec{e}^i_m]$.

##### Sequence learning network

When patient possesses multiple hospitalized visit records and needs to predict the treatment medications at current timestamp *t*, firstly, we require integrating described multi-sourced medical codes embedding vectors together at history timestamps, including the history diagnosis codes embedding representation sequence $\{\varvec{x}^1_d,\varvec{x}^2_d,\dots ,\varvec{x}^t_d\}$ and the history medication codes embedding representation sequence $\{\varvec{x}^1_m,\varvec{x}^2_m,\dots ,\varvec{x}^(t-1)_m\}$; afterwards, the temporal sequential learning network such as the recurrent neural networks (RNNs) are respectively utilized to capture the temporal dependencies of sequential medical codes as follows:

15 $$\begin{aligned} \varvec{h}^t_m&= \varvec{RNN}_{m}(\varvec{x}^1_m,\varvec{x}^2_m,\dots ,\varvec{x}^{t-1}_m),\nonumber \\ \varvec{h}^t_d&= \varvec{RNN}_{d}(\varvec{x}^1_d,\varvec{x}^2_d,\dots ,\varvec{x}^t_d). \end{aligned}$$

##### Medication prediction

The hidden state vectors $\varvec{h}^t_m$ and $\varvec{h}^t_d$ that incorporates the history information are obtained through the sequence learning network (Eq. (15)). However, considering the importance of current diagnosis information for the medication prediction at the current timestamp, the current diagnosis code embedding vector $\varvec{x}^t_d$ is also integrated into the patient representation. Therefore, the comprehensive patient representation vector $\varvec{O}_P$ can be calculated as follows:

16 $$\begin{aligned} \varvec{O}_P = \varvec{W}_P\cdot [\varvec{h}^t_m,\varvec{h}^t_d,\varvec{x}^t_d], \end{aligned}$$

where $\varvec{x}^t_d\in \mathbb {R}^{d+d_o+d_h}$, $\varvec{h}^t_m,\varvec{h}^t_d\in \mathbb {R}^{2d}$, and $\varvec{W}_P\in \mathbb {R}^{(5d+d_o+d_h)\times {d_e}}$ is the parameter to be learned. According to the comprehensive patient representation vector $\varvec{O}_P$, the current treatment medication $\hat{\varvec{y}}^m_{t}$ can be predicted as follows:

17 $$\begin{aligned} \hat{\varvec{y}}^m_{t} = \text {softmax}(\varvec{W}_O\cdot \varvec{O}_P+\varvec{b}_{o}), \end{aligned}$$

where $\hat{\varvec{y}}^m_{t}$ denotes the predicted multi-label medications set. $\varvec{W}_O\in \mathbb {R}^{{|\mathcal{C}_{m}|}\times {d_e}}$ and $\varvec{b}_{o}\in \mathbb {R}^{|\mathcal{C}_{m}|}$ are the parameters to be learned, where $\mathcal{C}_m$ denotes the medication codes set, and $|\mathcal{C}_m|$ is the size of set.

Due to the medication prediction task belonging to the domain of sequential multilabel prediction, we utilize the binary cross-entropy loss $\mathcal{L}$ as the objective function. According to the prediction result $\hat{\varvec{y}}^m_{t}$ at each timestamp *t* and the real label $\varvec{y}^m_{t}$, the predictive function binary cross-entropy loss is formulated as follows:

18 $$\begin{aligned} \mathcal{L}=-{\frac{1}{T-1}}\sum _{t=2}^{T} \varvec{y}^m_{t} \log \sigma \left( \hat{\varvec{y}}^m_{t}\right) +\left( 1-\varvec{y}^m_{t}\right) \log \left( 1-\sigma \left( \hat{\varvec{y}}^m_{t}\right) \right) \end{aligned}$$

## Experiments

### Experimental details

The experiment details include three parts: evaluation metrics, benchmark models and experimental setting.

#### Evaluation metrics

To evaluate the performance of the proposed KAMPNet, the Jaccard similarity score (Jaccard), precision-recall AUC (PR-AUC), and average F1 (F1) are adopted as the evaluation metrics. In practice, KAMPNet cannot wholly replace doctors and only screen possible medications as much as possible to assist physicians in prescribing medications for patients. Therefore, Jaccard should be one of the appropriate evaluation metrics. And it is defined as the size of the intersection divided by the size of the union of the predicted set $\hat{Y}_{t}^{m}$ and ground truth set $y_{t}^{m}$ as follows:

19 $$\begin{aligned} \text {Jaccard}=\frac{1}{\sum _{i}^{N} \sum _{t}^{T_{i}} 1} \sum _{i}^{N} \sum _{t}^{T_{i}} \frac{\left| Y_{t}^{i} \cap \hat{Y}_{t}^{i}\right| }{\left| Y_{t}^{i} \cup \hat{Y}_{t}^{i}\right| }, \end{aligned}$$

where $T_{i}$ is the number of visits for the $i^{th}$ patient, and *N* denotes the number of patients in the test set. And recall can be utilized to measure the predicted medications’ completeness:

20 $$\begin{aligned} \text {Recall}=\frac{1}{\sum _{i}^{N} \sum _{t}^{T_{i}} 1} \sum _{i}^{N} \sum _{t}^{T_{i}}\frac{\left| Y_{t}^{i} \cap \hat{Y}_{t}^{i}\right| }{\left| Y_{t}^{i}\right| } \end{aligned}$$

Furthermore, for the medication prediction task, due to the number of positive and negative labels being imbalanced, the precision-recall curve utilized to calculate the PR-AUC has proved to be an appropriate evaluation metric.

In addition, the predicted medications’ correctness should also be evaluated. Thus, the evaluation metric F1 is incorporated to evaluate the multilabel classification task comprehensively. Firstly, precision is generally adopted to measure the prediction correctness and can be calculated as follows:

21 $$\begin{aligned} \text {Precision}=\frac{1}{\sum _{i}^{N} \sum _{t}^{T_{i}} 1} \sum _{i}^{N} \sum _{t}^{T_{i}} \frac{\left| Y_{t}^{i} \cap \hat{Y}_{t}^{i}\right| }{\left| \hat{Y}_{t}^{i}\right| } \end{aligned}$$

Then, the metric F1 can be computed as follows:

22 $$\begin{aligned} \text {F1}=\frac{1}{\sum _{i}^{N} \sum _{t}^{T_{i}} 1} \sum _{i}^{N} \sum _{t}^{T_{i}} \frac{2\times \text {Precision}\times \text {Recall}}{\text {Precision} + \text {Recall}}. \end{aligned}$$

#### Benchmark models

To verify the superiority of the proposed model KAMPNet on medication prediction tasks, we compare it with the following baseline methods used for medication prediction, which include one machine learning-based method and six deep learning-based algorithms:

- *LR* [42]. It is a logistic regression with L1/L2 regularization. We sum the multi-hot vector of each visit together and apply the binary relevance technique [42] to handle the multilabel output.
- *Retain* [43]. RETAIN is an interpretable model with a two-level reverse time attention mechanism to predict diagnoses, which can detect significant past visits and associated clinical variables. It can be used for similar sequential prediction tasks, such as predicting treatment medicines.
- *LEAP* [13]. Leap formulates the medicine prediction problem as a multi-instance multilabel learning problem, mainly using a recurrent neural network (RNN) to recommend medicines.
- *GRAM* [7]. It utilizes the diagnosis ontology graph to enhance the diagnosis code representation by infusing the information from relational medical codes in a supervised method.
- *GAMENet* [4]. It employs a dynamic memory network to save encoded historical medication information, and further utilizes a query representation formed by encoding sequential diagnosis and procedure codes to retrieve medications from the memory bank.
- *G-BIRT* [5]. G-BIRT combines the graph neural network and bidirectional encoder representation from transformers (BERT) to enhance medical code representations through a pre-training method.
- *GATE* [31]. GATE constructs the dynamic co-occurrence graph at each admission record for every patient. It then introduces a graph-attention augmented sequential network to model the inherent structural and temporal information for medication prediction.

#### Experimental settings

To ensure the rationality of experimental verification, the pretreated clinical patients’ EMR data are still randomly divided into training, validation, and test sets with 2/3 : 1/6 : 1/6 ratios and the experimental results are the average values across five runs of random grouping and training. Moreover, the dimension of hidden layers and hyper-parameters in KAMPNet is set as follows: in the unsupervised graph contrastive learning framework for medical ontology graph, the dimension of hidden layers are set to 128, the node embedding size of graph encoder GAT is set to 32, the attention head is set to 4; in the unsupervised graph contrastive learning framework for medical codes relation graph, the dimension of hidden layers and the node embedding size of encoder GCN are all set to 64; the dimension of hidden layers in temporal sequential learning network is set to 256. In addition, the training is performed using Adam [44] at a learning rate of 5e-4, and we report the model performance in the test set within 40 epochs. All methods are trained on a Windows with 11GB memory and an Nvidia 2080Ti GPU using the deep learning computation platform Pytorch 1.6.

### Discussion

#### Discussion of prediction results

As demonstrated in Table 2, the treatment medication prediction results show that the performance of our proposed method KAMPNet is better than the existing state-of-the-art predictive models in health informatics in most cases. In detail, compared with baseline model LEAP, KAMPNet achieves about 10.52%, 9.46%, 13.4% higher performance concerning Jaccard, F1, and PR-AUC, respectively. We think the prominent reason might be that LEAP models the medication prediction problem as an instance-based medication prediction process which directly neglects the temporal dependency and does not consider the importance of implicit domain knowledge. The medication prediction performance of Retain is relatively better than LEAP, which could be attributed to its two-level attention-based model, which can capture the temporal relations and the relation between input features and output labels. Such a two-level attention-based sequential prediction model makes Retain’s performance even better than the GRAM model, which firstly introduces the hierarchical knowledge into the healthcare predictive model through the attention mechanism.

**Table 2:** Performance comparison on medication prediction task

Methods	Jaccard	F1	PR-AUC
$\text {LR}$	0.4075	0.5658	0.6716
$\text {LEAP}$	0.3921	0.5508	0.5855
$\text {Retain}$	0.4456	0.6064	0.6838
$\text {GRAM}$	0.4176	0.5788	0.6638
$\text {GAMENet}^{-}$	0.4401	0.5996	0.6672
$\text {GAMENet}$	0.4555	0.6126	0.6854
$\text {G-BIRT}$	0.4565	0.6152	0.6960
$\text {GATE}$	0.4742	0.6315	0.7087
$\text {KAMPNet}$	**0.4973**	**0.6454**	**0.7195**

Secondly, the similarity between GAMENet and KAMPNet is that the obtained diagnoses sequence and treatment medications sequence all utilize a sequence learning model to capture the temporal dependencies between medical codes. The difference is that GAMENet does not construct the relations between medical codes directly but constructs the medication-visit graph for capturing the indirect relations between medications for later retrieval using the attention mechanism. In other words, GAMENet considers modelling the co-occurrence relations between medications, while our KAMPNet takes the multiple relations between heterogeneous or homogeneous medical codes into consideration. Therefore, on the medication prediction task, our KAMPNet outperforms GAMENet by 4.18%, 3.28%, 3.41% on Jaccard, F1, PR-AUC, respectively. Unlike GAMENet, G-BIRT does not directly construct the relation between medical codes. Instead, it enhances the relation between medical codes using a pre-training method on the medical ontology graphs, including diagnosis code ontology graph and medication code ontology graph, which can make full use of the EMR data of single hospitalized patients. However, medical code embedding representation learning in G-BIRT mainly relies on the medication or diagnosis labels provided in history EMR and ignores the inherent relations between medical codes implied in EMR data. Although GATE also considers the relations between medical codes by building the co-occurrence graphs for each patient from the global guidance relation graph, it still neglects the infusion of valuable information of correlative medical codes from the medical codes relation graph and relies on the supervised label of the medication prediction task. Therefore, KAMPNet performs better than the latest knowledge-enhanced algorithm G-BIRT and GATE, and its Jaccard, F1 and PR-AUC improve at least by 2.31% on Jaccard, by 1.39% on F1 and by 1.08% on PR-AUC, respectively.

The critical reason that our proposed KAMPNet achieves the best performance compared with baseline models could be summarized as follows: (1) With the help of a multi-level unsupervised contrastive learning framework, it can capture the relations between medical codes and augments the medical codes representations based on the medical ontology graphs. (2) Then, the relations between medical codes implicit in the constructed medical code relations graph are further captured to learn more informative medical codes embedding representation vectors, contributing to the downstream tasks such as medication prediction. (3) The incorporated sequential learning network can further combine the supervised medical codes representations with the learned knowledge and relation augmented medical codes representations and then captures the temporal relations between medical codes for downstream medication prediction task.

#### Ablation study on model components

To verify the effectiveness of the critical components of KAMPNet and analyze their influence on the performance of medication prediction tasks, we conduct an ablation study to explore further the necessity of the proposed model components in our multi-level graph contrastive learning framework on the multi-sourced medical knowledge for the medication prediction task.

As illustrated in Table 3, compared with KAMPNet, the performance of five model variants decreased to varying degrees. We think the reason might be that the lack of model components leads to the failure of effective mining of the valuable relations between medical codes implicit in the multi-sourced medical knowledge. The details of the five variants are as follows:

- $\text {KAMPNet}_{{RG}^{-}}$. The variant $\text {KAMPNet}_{{RG}^{-}}$ does not consider the medical codes relation graph constructed based on the empirical knowledge from the history EMR data. That is, the relation augmented medical codes embedding representation vectors $\varvec{h}^i_d$ and $\varvec{h}^i_m$ are respectively excluded from the input of the corresponding sequence learning network. the performance of variant $\text {KAMPNet}_{{RG}^{-}}$ decreases by 1.48% on Jaccard, 1.01% on F1, and 0.22% on PR-AUC. The main reason is that the variant considers the locally inherent relations between medical codes in the medical ontology graph and does not consider capturing the valuable global relations between homogeneous or heterogeneous medical codes in the medical codes relation graph.
- $\text {KAMPNet}_{{HG}^{-}}$. The variant $\text {KAMPNet}_{{HG}^{-}}$ does not consider the medical domain knowledge graph, including the diagnosis code ontology graph and medication code ontology graph. That is, the knowledge augmented medical codes embedding representation vectors $\varvec{o}^i_d$ and $\varvec{o}^i_m$ are respectively excluded from the input of the corresponding sequence learning network. The performance of the variant declines by 1.81% on Jaccard, 1.41% on F1, 0.51% on PR-AUC, respectively, which is mainly because the medical codes relation graph can not acquire the meaningful initialization vectors for its nodes from the medical ontology graph. Moreover, the valuable relations between medical codes embodied in the inherent hierarchical structures in medical ontology graphs would not be captured for augmenting the medical codes embedding vectors.
- $\text {KAMPNet}_{{HGRG}^{-}}$. The variant $\text {KAMPNet}_{{HGRG}^{-}}$ concurrently neglects the multi-sourced knowledge augmented medical codes embedding representation vectors and considers the supervised learnable medical codes embedding representations $\varvec{e}^i_d$ and $\varvec{e}^i_m$ in a sequence learning network. It achieves lower by 1.64%, 1.29%, 0.57% than $\text {KAMPNet}$ respectively on Jaccard, F1, PR-AUC, but has relatively better performance than $\text {KAMPNet}_{{HG}^{-}}$, which demonstrates that there exists specific noise in the medical ontology graphs. In the future, we will explore how to effectively reduce the adverse effect of noise in multi-sourced medical knowledge.
- $\text {KAMPNet}_{R^{-}}$. The variant $\text {KAMPNet}_{R^{-}}$ indicates that the medical codes relation graph would not utilize the learned knowledge augmented medical codes embedding representation vectors to initialize the graph nodes (medical codes). It further validates the importance of such implicit information in the multi-level graph contrastive learning framework, which is proposed based on the relations between the medical ontology graph and the medical codes relation graph. It also indirectly explains why the performance of KAMPNet is relatively optimal when using the knowledge augmented medical codes embedding representation vectors as the initialization vector of the nodes of the medical codes relation graph.
- $\text {KAMPNet}_{{RG}^{W-}}$. The variant $\text {KAMPNet}_{{RG}^{W-}}$ considers whether there are relations between medical codes and does not consider the relation weights reflected in the medical codes relation graph. In this way, the performance of variant $\text {KAMPNet}_{{RG}^{W-}}$ decreases compared with presented $\text {KAMPNet}$, which further testifies that the relation weights based on co-occurrence probability obtained via the statistical method in the medical codes relation graph have a positive promoting effect on the performance of medication prediction.

**Table 3:** Performance comparison of the variants of KAMPNet on MIMIC-III dataset

Model	Jaccard	F1	PR-AUC
$\text {KAMPNet}_{{RG}^{-}}$	0.4825	0.6353	0.7173
$\text {KAMPNet}_{{HG}^{-}}$	0.4792	0.6313	0.7144
$\text {KAMPNet}_{{HGRG}^{-}}$	0.4809	0.6325	0.7138
$\text {KAMPNet}_{R^{-}}$	0.4866	0.6357	0.7147
$\text {KAMPNet}_{{RG}^{W-}}$	0.4911	0.6396	0.7165
$\text {KAMPNet}$	**0.4973**	**0.6454**	**0.7195**

Therefore, the proposed KAMPNet in this paper achieves the best performance only when the model’s components complement each other.

#### Analysis on the graph sparsity factor $\zeta$

In the construction process of the medical codes relation graph, considering the adverse effects of the noise introduced by relying solely on the statistical quantitative computation method and ignoring medical expertise, the graph sparsity factor $0<\zeta <PMI_{max}$ (Eq. 2) is incorporated in the Eq. 2. This section will explore the effect of graph sparsity factor $0<\zeta <PMI_{max}$ on medication prediction performance.

Table 4 shows the prediction results of treatment medications when the medical codes relation graph is constructed based on different graph sparsity factors. It can be seen from the table that when $\zeta =0.07$, the prediction performance of model KAMPNet is the best, and the performance decreases in varying degrees with the increase or decrease of $\zeta$ value. When $\zeta <0.07$, the redundancy of relations between medical codes might result in relatively more noise, which leads to a decline in prediction performance; when $\zeta >0.07$, with the increasing value of the graph sparsity factor, some meaningful edges with beneficial relation might be neglected due to the sparsity, which would bring about the incomplete captures of the relations between medical codes and would further cause the decline of model performance. In general, the value of the sparsity factor has a relatively small impact on the prediction results compared with the critical components studied in the Ablation study on model components section. The prominent reason may be that the noise influence in the medical codes relation graph is relatively small, and the valuable relations between medical codes and the augmentation of the embedding representation vectors of medical codes are still dominant. In addition, the final representation of medical code is concatenated by multi-source medical codes embedding representations. The incomplete learning of one source medical code embedding representation can not have a noticeable adverse impact on the prediction performance of the model KAMPNet. Factually, it further shows that the proposed KAMPNet is more robust in predicting treatment medications.

**Table 4:** The effect of graph sparsity factor $\zeta$ on model performance

$\zeta$	Jaccard	F1	PR-AUC
0.01	0.4917	0.6398	0.7176
0.02	0.4908	0.6392	0.7163
0.03	0.4895	0.6384	0.7187
0.04	0.4948	0.6431	0.7199
0.05	0.4916	0.6407	0.7176
0.06	0.4924	0.6407	0.7196
0.07	**0.4973**	**0.6454**	0.7195
0.08	0.4922	0.6404	**0.7201**
0.09	0.4933	0.6413	0.7194
0.10	0.4903	0.6382	0.7175

#### Effects of graph encoders in contrastive learning networks

The multi-level unsupervised contrastive learning framework described in Materials and methods section, including the medical domain knowledge graph-based contrastive learning framework and the medical codes relation graph-based unsupervised contrastive learning framework, possesses respectively different graph encoders because of distinct reasons. In this section, we will analyze the selection of different graph encoders in the graph contrastive learning framework and explore their effects on downstream tasks such as medication prediction results. In Table 5, “Encoder in HG” indicates the applied graph encoder in the contrastive learning framework based on the medical domain knowledge graph (HG), while “Encoder in RG” represents the applied graph encoder in the contrastive learning on the medical codes relation graph (RG). $\surd$ denotes using the corresponding graph encoder, while $\times$ denotes not using the corresponding graph encoder.

**Table 5:** The effect on prediction performance of different graph encoders

Model	Encoder in HG		Encoder in RG		Prediction Performance		
	GAT	GCN	GAT	GCN	Jaccard	F1	PR-AUC
$\text {KAMPNet}_{AC}$	✓	✗	✗	✓	0.4973	0.6454	0.7195
$\text {KAMPNet}_{AA}$	✓	✗	✓	✗	0.4878	0.6381	0.7160
$\text {KAMPNet}_{CC}$	✗	✓	✗	✓	0.4869	0.6373	0.7176
$\text {KAMPNet}_{CA}$	✗	✓	✓	✗	0.4853	0.6358	0.7122

$\text {KAMPNet}_{AC}$ indicates that the graph encoders in HG and RG based contrastive learning frameworks are graph attention network (GAT) and weighted graph convolutional network (GCN), respectively, which achieves the relatively optimal prediction result. While in $\text {KAMPNet}_{CC}$, the graph encoder in HG based contrastive learning framework is replaced with general GCN, which results in a decrease in the medication prediction performance. We think the main reason is that GAT can aggregate the information of correlative neighbourhood codes to the leaf codes according to the learned relevance scores between connected medical codes, while general GCN does not consider the relation weights between medical codes and aggregate the information of connected (or correlative) medical codes equally. Compared with variant $\text {KAMPNet}_{AC}$, variant $\text {KAMPNet}_{AA}$ directly uses GAT as the graph encoder, which could relearn the relevance score and does not consider the relation weights representing the empirical knowledge from medical codes relation graph. The result is a decrease in prediction performance. Using GAT as the graph encoder in the medical codes relation graph-based contrastive learning framework is not appropriate. We think the main reason is that the learned normalized relation score between medical codes in GAT belongs to an uncertain relevance score. In contrast, the empirical relation weight between medical codes computed based on the co-occurrence probability in the medical codes relation graph is a relatively specific relevance score. Therefore, compared to the above other variants, $\text {KAMPNet}_{CA}$ performs worst on the medication prediction task.

#### Limitations of the medication prediction model

Though the above extensive experiments have testified the efficacy of KAMPNet for reasonable medication prediction, there are still some limitations on the results due to the complexity of the healthcare system. First, the real clinical decision-making process is full of uncertainty caused by various factors such as the professional level of doctors, the social environment, and the economic conditions of patients, which might cause medication prediction bias. Thus, in the future, we would consider cultivating more advanced methods to reduce the impact of bias on the prediction outcomes. Second, the medication prediction model is essential for hospitals, clinics, and retail pharmacies. Since we only utilized the MIMIC-III dataset to testify the efficacy of our proposed model, the prediction outcomes of the model are not guaranteed on the data from other sources. However, provided the data has similar composition and structure defined in the previous section, the model should be applicable after fine-tuning. In the future, we will further evaluate our model on the datasets from clinics or retail pharmacies.

Additionally, the use of machine learning models in healthcare presents several ethical implications, such as privacy and data security, transparency and interpretability, informed consent and autonomy, human oversight and decision-making, etc. And here are some corresponding potential approaches to address them: (1) Cultivate encryption techniques and secure data storage protocols to safeguard sensitive data; (2) Develop explainable and interpretable machine learning models that provide insights into the factors influencing predictions; (3) Educate patients about the use of machine learning models in their healthcare and provide clear explanations of the benefits, risks, and limitations; (4) Offer patients the option to opt out of using machine learning models in their treatment decisions if they have concerns or preferences; (5) Encourage interdisciplinary collaboration between healthcare providers and data scientists to ensure a holistic approach to patient care. Thus, in the future, collaboration, transparency, and an ongoing commitment to ethical practices are essential to ensure the responsible and beneficial use of machine learning models in healthcare.

For the practical applications of model KAMPNet in the future, clinically, it could be utilized to assist doctors in making informed medication decisions for patients according to electronic medical records (EMR). In addition, the model could also be used to assist the hospital pharmacy management department, clinic, or retail pharmacies to predict the medications in advance for stocking. However, the model needs to be retrained using the datasets from different application scenarios. Thus, in the future, a user study pertaining to the practical application scenario should be undertaken for validating the model’s feasibility in practice. Additionally, owing to the limited size of the population cohort itself, and the experimental outcomes of existing approaches, the effect of the size of experimental samples on the prediction outcomes is neglected in our manuscript. In the future, the above factors will be taken into account once applied in the new dataset or private dataset for the generalization.

## Conclusion and future work

In this study, we propose a multi-sourced medical knowledge-augmented medication prediction network. Expressly, we incorporate a novel multi-level graph contrastive learning framework for fully capturing the valuable relations between medical codes implicit in the multi-sourced medical knowledge. The framework firstly leverages the local graph contrastive learning on the medical ontology graphs to learn the knowledge augmented embedding vectors of diagnosis codes and medication codes, which factually have infused the information of correlative medical codes into each other in the learning process. Then, the medical codes relation graph is constructed and utilized to learn the relation augmented medical codes embedding vectors using the graph contrastive learning framework, which aims to capture the global relations between homogeneous and heterogeneous medical codes. Finally, the multi-channel sequence learning network is presented to capture the temporal relations between medical codes, by which we can get a comprehensive patient representation for downstream tasks such as medication prediction. We evaluate the performance of the proposed KAMPNet on a real-world clinical dataset, and the experimental results show that our model achieves the best medication prediction performance against baseline models in terms of Jaccard, F1 score, and PR-AUC.

With the help of the presented framework, in the future, we can introduce more related medical domain knowledge, such as the medication-related molecular graph and the bipartite graph representing adverse medication reactions. In addition, we will cultivate more advanced algorithms to better mine the insightful information implicit in the multi-sourced medical knowledge and explore how to determine the importance of multi-sourced medical knowledge automatically.

## Data Availability

The data used in the paper is from a publicly available dataset MIMIC-III [35]. The processed data can be obtained on Github (https://github.com/uctoronto/KAMPNet/tree/main/Data).

## Abbreviations

KAMPNet: Knowledge augmented medication prediction network
EMR: Electronic medical records
ICD: International classification of diseases
ATC: Anatomical therapeutic chemical classification system
MIMIC: Medical information mart for intensive care
BERT: bidirectional encoder representation from transformers
DGI: Deep graph infomax
LSTM: Long short-term memory
GCN: Graph convolutional network
GNN: Graph neural network
GAT: Graph attention network
LR: Logistic regression
LEAP: Learn to prescribe
GRAM: Graph-based attention model
GAMENet: Graph augmented memory networks
GATE: Graph-Attention augmented temporal neural network
RETAIN: Reverse time attention
